# The Comparative Effectiveness of Virtual Reality Versus E-Module on the Training of Donning and Doffing Personal Protective Equipment: A Randomized, Simulation-Based Educational Study

**DOI:** 10.7759/cureus.23655

**Published:** 2022-03-30

**Authors:** Meryl B Kravitz, Nicholas B Dadario, Adeel Arif, Simon Bellido, Amber Arif, Oark Ahmed, Marc Gibber, Farrukh N Jafri

**Affiliations:** 1 Otorhinolaryngology-Head and Neck Surgery, Montefiore Medical Center, Wakefield Campus, Bronx, USA; 2 Medicine, Robert Wood Johnson Medical School, Rutgers University, New Brunswick, USA; 3 Simulation Laboratory, White Plains Hospital, White Plains, USA; 4 Medicine, State University of New York (SUNY) Downstate Medical Center, Brooklyn, USA; 5 Emergency Medicine, Montefiore Medical Center, Wakefield Campus, Bronx, USA; 6 Emergency Medicine, White Plains Hospital, White Plains, USA

**Keywords:** virtual reality in medical education, simulation in medical education, donning and doffing, personal protective equipment (ppe), virtual reality simulation

## Abstract

Introduction

Preventing errors in donning and doffing of personal protective equipment (PPE) is critical for limiting the spread of infectious diseases. Virtual reality (VR) has demonstrated itself as an effective tool for asynchronous learning, but its use in PPE training has not been tested. The objective of this study was to compare donning and doffing performance between VR and e-module PPE training.

Methods

A prospective randomized open-blinded controlled trial was conducted to determine differences in donning and doffing performance after VR and e-module PPE training among medical staff and medical students at a single institution. The primary outcome was donning and doffing performance with real PPE, assessed using a 64-point checklist. The secondary outcome was participant preparedness and confidence level after training.

Results

Fifty-four participants were randomized, mostly consisting of medical students (n=24 {44%}) or emergency medicine and otolaryngology residents (n=19 {35%}). The VR group (n=27 {50%}) performed better than the control in the overall PPE scores but this was not statistically significant (mean {SD}, VR: 55.4 {4.4} vs e-module: 53.3 {8.1}; p = 0.40). VR participants also reported higher levels of preparedness and confidence after training. Residents as a subgroup achieved the highest increases after VR training compared to their counterparts in the control training group (mean {SD}, VR: 55.6 {4.9} vs e-module 48.4 {5.5}, p = 0.009).

Conclusion

In this randomized trial, VR training was found to be non-inferior to e-module for asynchronous PPE training. Our results suggest that in particular residents may benefit most from VR PPE training. Additionally, VR participants felt more confident and prepared to don and doff PPE after training compared to e-module participants. These findings are particularly relevant given the ongoing coronavirus disease 2019 (COVID-19) pandemic. Future studies need to focus on VR integration into residency curriculum and monitoring for long-term skill retention.

## Introduction

The coronavirus disease 2019 (COVID-19) pandemic highlighted concerns regarding personal protective equipment (PPE) utilization in hospitals [1,2]. When used correctly, PPE can minimize transmission to COVID-19 [3,4]. However, evidence suggests that up to 90% of PPE procedures like doffing are performed incorrectly [5]. This failure rate has been linked to healthcare workers being more likely to contract COVID-19 compared to the general community, resulting in authorities turning to increased training as a potential solution [6,7].

PPE training is mandated for all front-line healthcare workers in the United States, yet there is no gold standard for doing so [8]. Training methods vary, with the conventional approaches being in-person or video presentations [8]. In-person, immersive training with active involvement and feedback tends to be preferred; however, staff shortages, PPE shortages, and social distancing guidelines limit feasibility [9,10]. Online modules and videos are asynchronous methods that are also commonly utilized, but pose problems including lack of student engagement, reduced accountability, and the limitations of teaching hands-on skills online [11]. 

Virtual reality (VR) is a potential alternative, offering similar benefits to in-person training, such as immersion and feedback, while minimizing barriers related to timing, social distancing, and equipment shortages [12]. VR allows for repetitive practice on an as-needed basis while preserving PPE for clinical interactions. Further, VR headsets continue to grow in availability and affordability. These qualities make VR a viable alternative, although its impact on donning and doffing quality is unknown. Studies regarding PPE training have found in-person and video methods to be comparable [11,13], and computer simulations effectively complement in-person training [14]. However, to our knowledge, there has been no investigation of the use of VR for PPE training.

This study conducted a randomized clinical trial at a tertiary care academic center to evaluate the effectiveness of VR training compared to a control group consisting of electronic-module (e-module) training as it relates to donning and doffing competency. The primary objective was to compare the performance of donning and doffing PPE after VR or control. Secondary objectives included identifying subgroups more likely to benefit from the training and evaluating participants' perceptions of using of VR for PPE training. It was hypothesized that the use of VR training will be superior to the e-module as an asynchronous modality to teach PPE procedures.

## Materials and methods

This study was a parallel, 1:1 randomized trial on a convenience sample conducted at an urban academic medical center with a prospective open blinded endpoint (PROBE) design [15]. The Consolidated Standards of Reporting Trials (CONSORT) were followed [16]. The study was approved by the institutional review board (IRB); verbal and signed consent was obtained from all participants.

Participants

Members of the institution were included. Participants were recruited using email listservs, and the study was primarily conducted at the hospital's simulation center. To meet the needs of participants, we expanded to multiple locations, including residency conferences for emergency medicine and otolaryngology. These residencies were chosen due to invitation from residency directors and their increased risk for infectious exposure during the pandemic.

Randomization

**Figure 1:**
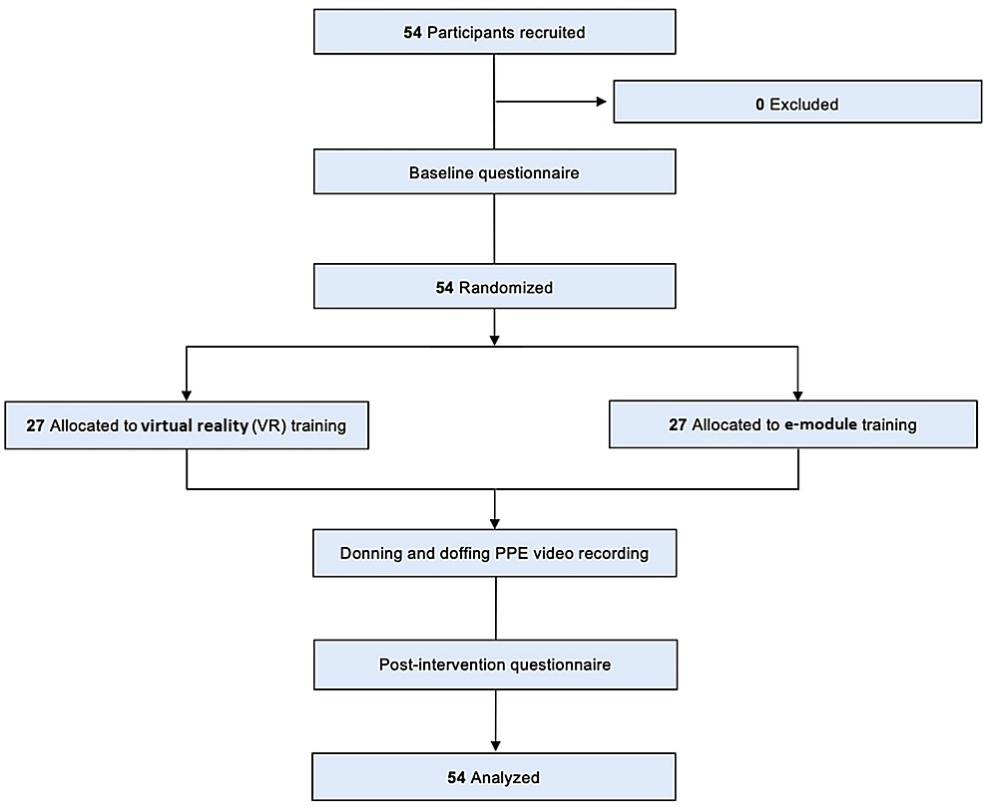
Study flow diagram of control (e-module) and intervention (VR) groups. PPE: personal protective equipment

Upon enrollment, participants were assigned an identification study number and were block randomized using a concealed computer-generated random allocation sequence. Randomization was performed on-site in real-time. Participants were separated into control (e-module) or intervention (VR) groups (Figure 1). After providing consent, participants completed a short anonymous survey regarding demographics, PPE training, and VR experience. 

Control

The control group received online PPE training that was completed at the study center. Training included a five-minute instructional video and a PowerPoint presentation. The video included step-by-step instructions and a demonstration of donning and doffing. The presentation contained the same content as the home institution’s PPE training, based on Centers for Disease Control and Prevention (CDC) guidelines [7]. Participants were instructed to review the material however they liked, and the duration of their training was recorded. A study member was also present for any assistance that was needed with the PPE training e-module. 

Intervention

**Figure 2:**
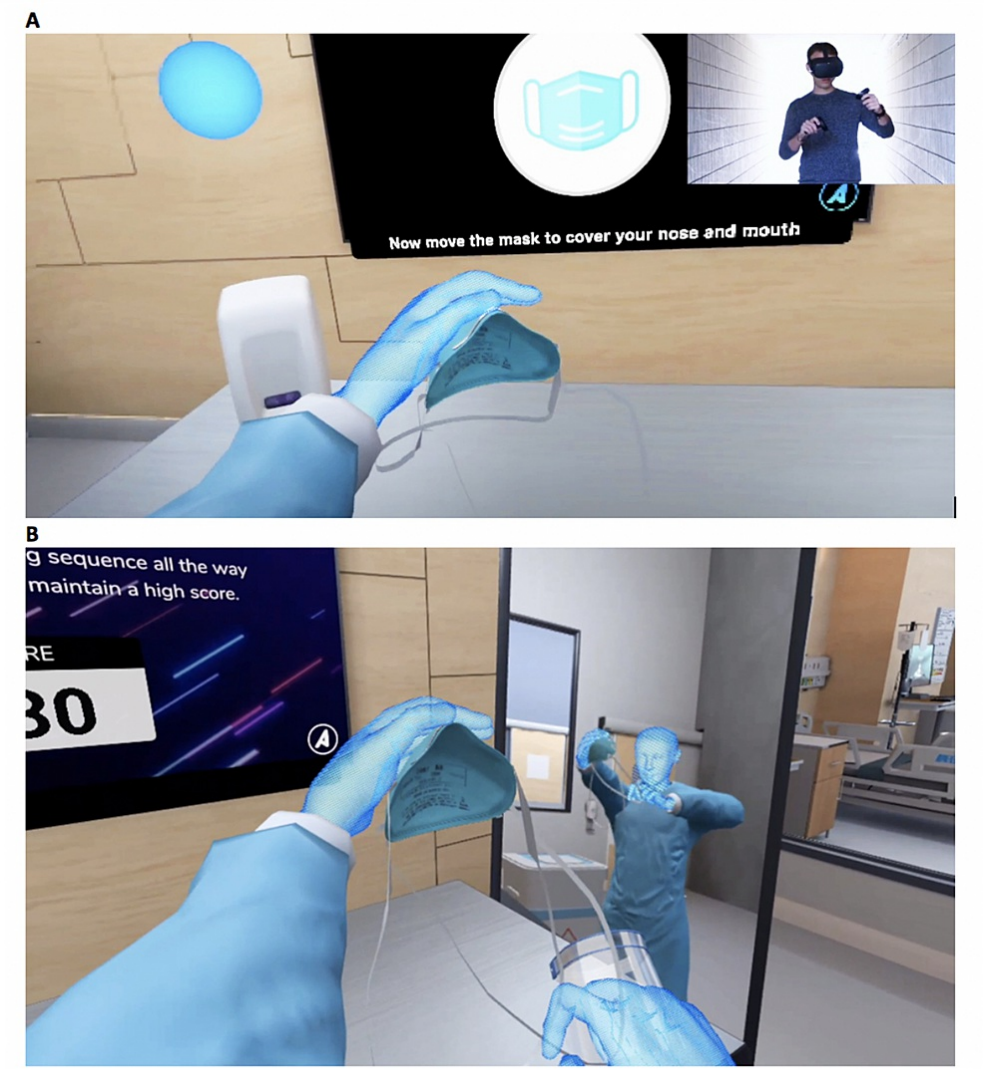
Training mode allows for guided, repetitive practice of donning and doffing (A). In testing mode, users demonstrate their skills for a score (B).

The intervention group received VR-based PPE training on the Oculus Quest (Menlo Park, CA: Facebook, Inc.) using a PPE training program created by Axonpark, Inc. (Fort Lauderdale, FL). The training included the following: (1) a tutorial of the donning and doffing sequence, based on CDC guidelines [7]; (2) a training mode to practice with stepwise feedback; and (3) a testing mode that repeated until the sequence was completed perfectly (Figure 2, Video 1). A study member was present to assist with the device and record the duration of training. No affiliate of Axonpark, Inc. was present during the course of the study. 

 

**Video 1:** VR-based PPE training created by Axonpark, Inc. The VR training includes three distinct modes which focus on stepwise feedback and Rapid-Cycle Deliberate Practice. PPE: personal protective equipment; VR: virtual reality

Skill assessment

Donning and doffing performance was assessed using videos of participants with real PPE. After completing training, participants were brought to a private, recorded room with the necessary PPE and instructed to don and doff. Finally, participants completed a survey regarding reactions to the program, physical complaints, and feedback. 

Outcomes

The primary outcome measure was donning and doffing performance - each measured separately - which was evaluated using a checklist based on CDC guidelines (Table 1) [7]. Each step represented a concept from training, albeit with varying degrees of contamination risk. To address this, steps were assigned as major (four points) or minor (two points). Further, as sequence is a critical component of donning and doffing, steps completed off sequence were awarded half credit. Missed steps were awarded zero credit. Total possible score was 36 for donning and 28 for doffing, for a sum of 64 for the overall PPE score. Secondary outcome measures included subgroup analysis and survey data such as participants’ perceived preparedness, perceived confidence, and degree of distraction during training. 

**Table 1:** Checklist for donning and doffing

Donning	S. no.	Correct sequence
Major	1	Sanitize hands before gowning
Major	2	Open gown and inspect
Minor	3	Put arms down sleeves of gown
Major	4	Secure neck strap of gown before waist strap of gown
Major	5	Put on mask
Minor	6	Secure bottom strap of mask before top strap of mask
Major	7	Adjust nose piece
Major	8	Put on face shield
Major	9	Sanitize hands before putting on gloves
Major	10	Put on gloves
Minor	11	Pull gloves over cuff of gown, if not already over
Doffing	S. no.	Correct sequence
Major	1	Sanitize hands with gloves still on
Major	2	Pull gown forward, break straps on back and neck (considered an error if they untie gown instead of ripping)
Minor	3	Remove gown and gloves together in one shot (considered an error if they remove gown and glove is still on. If this is the case, please document)
Major	4	Sanitize hands before doffing face shield
Major	5	Remove face shield touching the back only
Major	6	Sanitize before doffing the mask
Minor	7	Remove the bottom strap of the mask before removing top strap of mask
Major	8	Sanitize hands

Raters

Due to the nature of the intervention, participants were not blinded, but outcome raters were. The outcome raters were two independent nurse educators, blinded to the study protocol and interventions. Raters were calibrated using a standardized scoring checklist and assessed through ratings of mock videos. To assess the PPE performance of participants, raters were randomly assigned 35 videos each, with a 16-video overlap to calculate reliability. Recordings were stored in an encrypted and secure folder.

Statistical analysis

All statistical analyses were performed in R version 3.6.3 (Boston, MA). Following data collection, comparisons between study groups were examined at an alpha level = 0.05. The main outcome was the continuous outcome of “overall score” - the summation of donning and doffing scores. A two-way intraclass correlation (ICC) analysis of both rater scores assessed for agreement and consistency in grading both primary and secondary outcomes [17]. Univariate analyses and chi-squared tests with Yates’ continuity correction of all demographic variables and study groups determined potential associations and the efficacy of randomization. All continuous outcomes were compared with unpaired t-tests, analysis of variance (ANOVA), or Wilcoxon rank-sum tests based on whether they met appropriate statistical assumptions. Multivariate regression analyses of the primary outcome controlled for demographic associations. Subgroup analyses determined the possible effects of the interventions on the heterogeneous sample.

Ordinal logistic regressions determined the log odds likelihood of reporting different levels of Likert-type survey data. Corrected chi-squared tests compared reported distractibility between groups. The VR group was further analyzed regarding their experience with the VR platform.

## Results

The study was conducted from November 16, 2020, to January 27, 2021. Fifty-four participants provided informed consent and were randomized: 27 (50%) to the VR group and 27 (50%) to the e-module group. Outcome assessment was performed in 54 (100%) of all participants (Figure 1). 

Demographics

Participants were on average 30.5 years old (SD = 8.6), female (63%, n = 34), and primarily medical students (44%, n = 24) or residents (35%, n = 19). Most had no previous PPE experience (52%, n = 28) or clinical experience (46%, n = 25). Prior PPE training in the previous year mostly consisted of videos (n = 9). Most had no previous experience with VR (n = 33), but the VR group had more VR experience than the e-module group (59% vs 26%, p = 0.03). Both groups reported a similar median (SD) level of fear about previous contamination (VR = 3 {0.96} vs. e-module = 3 {1.18} = 3) (Table 2). 

**Table 2:** List of demographic data in virtual reality (VR) and e-module (control) study groups. There were no statistically significant differences (p < 0.05) between study groups in each demographic variable as assessed with Pearson’s chi-squared test with Yates’ continuity correction and univariate regression analyses. PPE: personal protective equipment

Characteristic	Virtual reality (n = 27)	E-module (n = 27)
	No. (%)	No. (%)
Age, mean (SD)	31.4 (8.6)	29.6 (7.4)
Female sex	17 (63)	17 (63)
Prior PPE training, mean	12 (44)	14 (52)
> 1 year ago	5 (19)	10 (37)
0-12 months ago	12 (44)	10 (37)
Never	10 (37)	8 (30)
Prior clinical experience, mean (SD)	4.0 years (SD=10.57)	2.6 years (SD = 6.57)
10+ years	2 (7.4)	2 (7.4)
1-10 years	13 (48)	12 (44)
None	12 (44)	13 (48)
Occupation		
Medical student	11 (41)	13 (48)
Resident	10 (37)	9 (33)
Attending physician	1 (3.7)	2 (7.4)
Paramedic	2 (7.4)	0 (0)
Registered nurse	1 (3.7)	0 (0)
Research associate	1 (3.7)	1 (3.7)
Simulation specialist	0 (0)	1 (3.7)

Overall donning and doffing PPE score

**Figure 3:**
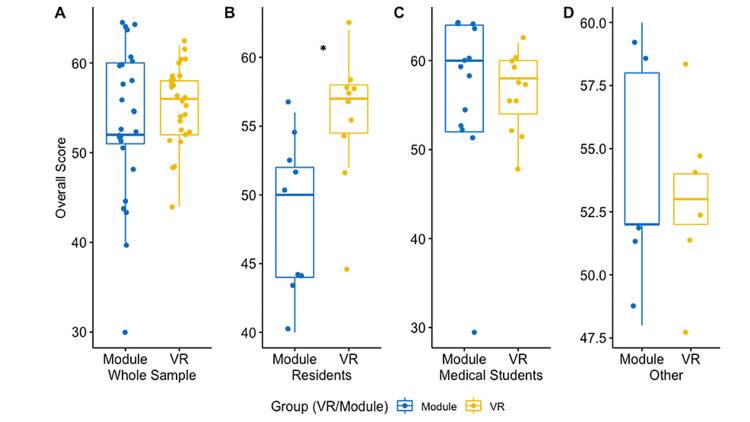
Visual comparison of the average performance in overall PPE score between virtual reality (VR) and control (module) groups. Scores are represented for the whole study sample (A), residents alone (n = 19) (B), medical students alone (n = 24) (C), and all other participants (n = 9) (D). Residents in the VR group on average demonstrated higher overall PPE scores compared to non-residents in the VR group. *P-value < 0.05. PPE: personal protective equipment

The primary outcome was overall donning and doffing PPE score and was assessed with the Wilcoxon rank-sum test with continuity correction. The VR group had higher, but non-significant, scores than the e-module group (55.4 {SD = 4.4} vs 53.3 {SD=8.1}, p = 0.40, 95% CI = -6.00 to 2.00) (Figure 3). 

When assessing the individual components of donning and doffing, scores had non-normal distributions and were examined with corrected Wilcoxon rank-sum tests. VR had non-significant increases compared to the e-module in both donning and doffing scores (donning = 30.1 {3.3} vs 29.7 {4.9}, p = 0.94, 95% CI = -2.00 to 2.00; doffing = 25.3 {2.4} vs 23.6 {4.8}, p = 0.26, 95% CI = -4.54e-0.5 to 4.00).

Fisher’s exact test assessed the amount of major or minor steps completed correctly between groups. VR demonstrated significantly more steps that were correctly performed in regard to major doffing steps and minor donning steps (p < 0.05). Similar favorable, but non-significant, trends can be seen for all other categories. Time spent in training was measured with a Wilcoxon rank-sum test and found that the VR group spent significantly more time in training than the e-module (VR, 25.6 min vs e-module, 6.5 min; p < 0.001; 95% CI = -20.0 to 16).

Subgroup analyses

Subgroup analyses were based on the most common occupations listed. The study sample was reduced into three groups: medical students, residents, and all other participants (Table 3). The primary outcome was analyzed in medical students with a Wilcoxon rank-sum test with continuity correction and in other groups with a Welch two-sample t-test. In medical students, there was no significant difference between study groups (VR, 56.55 vs e-module, 56.46; p = 0.98; 95% CI = -6.15 to 5.98). In residents, those utilizing VR performed superiorly to those utilizing e-module (VR, 55.6 vs e-module, 48.4; p = 0.01; 95% CI = 2.05-12.26). All other participants demonstrated no significant difference between study groups (VR, 53.0 vs e-module, 54.0; p = 0.93; 95% CI = -6.00 to 8.00).

**Table 3:** Overall PPE score performance regression analysis. Regression analysis modeling possible predictor variables, including study group and resident status, on overall PPE scores. Robust regression was utilized to calculate robust standard errors with the ("rlm" command and sandwich package in R) on our outcomes of interest. Reference groups used for each variable are listed in the table columns. Previous PPE training or clinical experience were created as dichotomous variables (1 = yes or 0 = no). No main effect of group on overall PPE score was demonstrated. However, a significant interaction was demonstrated between status as a resident and the study group on overall PPE scores. These data demonstrate that VR residents were superior to control group residents. *P-value < 0.05. **P-value < 0.001. PPE: personal protective equipment

Overall PPE score performance				
Predictors	Estimate	Standard error	t-Value	p-Value
(Intercept)	59.71	4.29	13.92	2.2e-16**
Group (reference: VR)	0.02	2.31	0.01	0.99
Resident status (reference: current resident)	-7.37	2.733	-2.70	0.01*
Group resident status (interaction)*	6.98	3.33	2.10	0.04*
Age	-0.11	0.13	-0.88	0.39
Gender (reference: male)	-2.91	2.09	-1.39	0.17
Previous PPE training	1.06	1.74	0.61	0.55
Previous clinical experience	0.02	0.10	0.23	0.82

**Figure 4:**
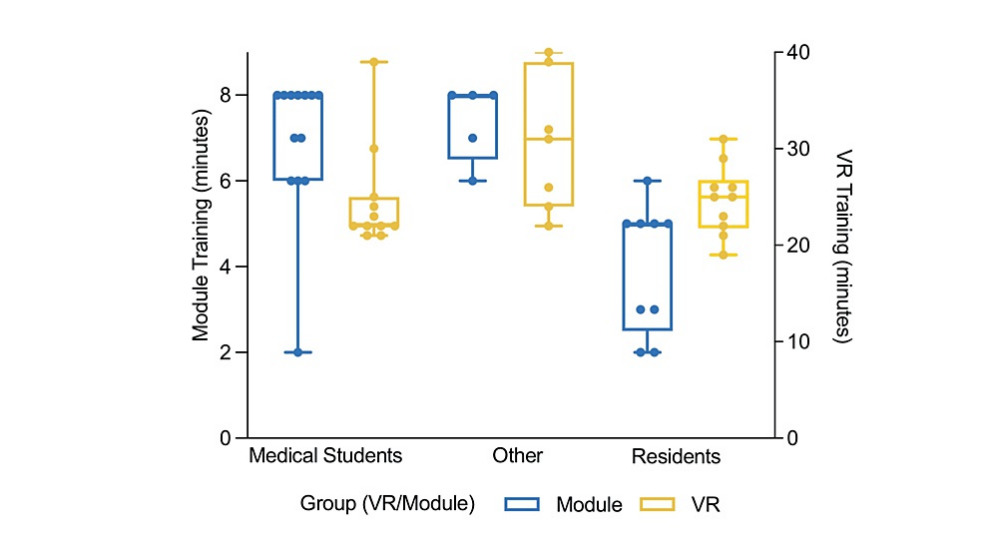
Training time differences among subgroups Visual comparison of the average training time among subgroups between virtual reality (VR) and control (e-module) groups. Residents in the e-module group spent significantly less time training compared to medical students and other participants (p < 0.05). However, there was no significant difference in VR training time among the three subgroups.

Differences in training time were measured with a one-way analysis of variance (ANOVA). There was a significant difference in e-module training time for residents (M = 4.0 min, SD = 3.52), medical students (M = 6.9 min, SD = 1.7), and other participants (M = 7.4, SD = 0.9) (F{2,24} = 12.15, p = 0.0002). Post hoc comparisons using the Tukey's Multiple Comparison test indicated that the mean training time for residents was significantly different than for medical students (p = 0.0005, 95% CI = 1.27-4.58) and other participants (p = 0.0015, 95% CI = 1.27-5.53). However, there was no significant difference between e-module training time for medical students and other participants (p = 0.83). Alternatively, a one-way ANOVA in VR training time among all three groups found no significant differences (F{2,25} = 3.2, p = 0.31) (Figure 4).

In the resident group, doffing and donning scores were analyzed with a corrected Wilcoxon rank-sum test. Residents utilizing VR achieved higher doffing scores than those utilizing the e-module (26.0 {SD = 2.7} vs 21.8 {SD=4.3}, p = 0.02, 95% CI = 0.64-7.81). Similarly, the VR group achieved higher, but non-significant, donning scores compared to the e-module group (29.6 {SD = 2.6} vs 26.6 {SD = 4.4}, p = 0.14, 95% CI = -8.00 to 2.00). 

An additional variable was created for status as a resident for regression analyses. A robust regression model controlled for age, gender, PPE experience, clinical experience, and status as a resident. Interaction effects were analyzed between status as a resident and the study group [18]. A significant two-way interaction was identified between resident status and study group on the overall score. Residents in the VR group scored 6.98 points higher than residents in the e-module group (p = 0.04). However, for non-residents, there was no difference between VR and e-module groups (p = 0.99) (Table 3).

Participant experience

Ordinal regression of survey responses measured perceived preparedness and confidence retaining information after training. VR was superior to e-module in both outcomes. In terms of perceived preparedness, log odds of reporting a lower score of 3 compared to 4 or 5 is 1.08 points lower in VR than e-module (p = 0.05). For perceived confidence of retention, the log odds of reporting a lower score of 3 compared to 4 or 5 is 1.55 points lower in VR than for e-module (p = 0.007). A larger, but non-significant, percentage of the E-module group reported being distracted compared to VR (59% vs 48%, p = 0.58).

Rater scores

The raters demonstrated strong agreement and consistency in primary and secondary outcomes. In overall PPE score, the two-way ICC score for agreement was 0.88 (p < 0.001, 95% CI = 0.64-0.96) and consistency was 0.89 (p < 0.001, 95% CI = 0.78-0.96). Strong agreement and consistency were also demonstrated in the donning score (agreement = 0.84, p = 0.003; consistency = 0.86, p = 0.002) and doffing score (agreement = 0.92, p < 0.001; consistency = 0.92, p < 0.001).

Feedback on VR platform

No participants in the VR group reported experiencing motion sickness (n = 27). Physical complaints reported included blurry vision due to fogging (n=4), claustrophobia (n=1), and headache (n=1). No participants in the e-module group reported physical complaints. In the VR group, eight participants experienced minor technology malfunctions requiring technical assistance, including repeated attempts to register steps (n=5), random restart (n=2), and defective sound from the device (n=1).

## Discussion

In this randomized, intervention-controlled trial, VR training was found to be non-inferior to e-module training, although VR participants reported statistically significant increases in preparedness and confidence in donning and doffing PPE after training.

The study population was heterogeneous and the effects of the intervention differed depending on occupation - importantly, residents compared to non-residents. Residents utilizing VR achieved statistically significant increases in PPE score compared to those utilizing e-modules, while non-residents had non-significant increases in VR compared to e-module groups. These observations likely result from differences in training time between residents and non-residents in the e-module group, namely that the residents in the e-module group spent significantly less time in PPE training and thus obtained decreased PPE scores compared to other participants utilizing the e-module (Figure 3). On the contrary, residents in the VR group spent just as much time as medical students in training, lending itself to PPE scores comparable to other participants.

Nonetheless, this study’s findings confirm the potential of e-module training in certain motivated groups but suggest it may be less effective with other individuals [19]. Residents represent an important subgroup with regards to PPE competency, as many were deployed to the front lines during the initial COVID-19 response and may have experienced some of the highest risks for exposure [20]. At this very institution, a study found that 42% of internal medicine residents deployed during the initial COVID-19 response presented with COVID-19 or COVID-like illness [21]. This rate is likely comparable to emergency medicine and otolaryngology residents, who held similar roles at the time. Of note, while 68% of residents in the study reported treating more than 40 COVID-19 patients in the last year, 47% of residents reported never receiving PPE training and 21% of residents had it more than one year prior, highlighting the importance of finding the right training for the right population at the right time. 

Although the results of this study cannot support the hypothesis that VR PPE training is superior to e-module, they do suggest that VR may be an effective training tool in specific groups, such as residents. Several factors may explain this effect. Most importantly, residents may have had difficulty engaging with the e-module, as shown by the significantly decreased e-module training time in residents compared to medical students versus the similar training times between these two groups in VR training. Increased stressors during residency may influence the quality of learning devoted to self-paced instruction [22]. Residents may be more susceptible to distractions in non-immersive environments, and stressors can encourage quicker completion of training [23]. Additionally, given the experimental design, residents may have been less vulnerable to the Hawthorne effect compared to their medical student counterparts [24]. At earlier stages of their careers, medical students may consciously or subconsciously perform differently in the presence of superiors. Therefore, resident effort and time towards e-module training may be more realistic to the manner e-modules are completed without supervision. Finally, residents who went through a more engaging and interactive simulation-based environment (e.g., VR training) may have performed significantly better due to the high correlation between learner engagement and the effectiveness of teaching [25]. It has been found that engaging methods of learning can help maximize retention, decision making, and psychomotor skills of medical tasks - each of which is critical in stressful emergency step-by-step procedures like PPE donning and doffing [26-28]. 

Limitations and future directions

The current study recruited from a heterogeneous convenience sample of participants within a single hospital system, and selection bias may have influenced results. As a single institution with already limited study power, it was not feasible to execute numerous multi-factorial randomized studies to study this intervention in separate groups. Additionally, given the recent pandemic, there was an urgent need for healthcare workers to learn donning and doffing. Therefore, to understand which populations benefit from VR-based training and also control for selection biases, numerous subgroup analyses were conducted. 

An inherent limitation of most VR-based programs such as our own is the additional time required to complete the necessary training. VR programs include additional tutorial and training features that do not allow skipping through materials, but such features may actually provide additional benefits in the retention of the learned material [29]. 

Additionally, both training modalities were not true asynchronous modes of learning since the research team was present to assist the participants if needed. The participants of the VR group had more assistance than the e-module since the VR game had more technological issues. Further studies should evaluate these modalities in a more asynchronous setting. 

Lastly, the donning and doffing performance checklist, although based on CDC guidelines, has not been validated. To our knowledge, there is only one validated rubric from 2014, which does not emphasize the correct sequence of steps [30]. The research team worked with selected experts to create a performance checklist that prioritized sequence and allowed for analysis of individual steps as well as general performance. This checklist allowed for rigorous analysis of PPE performance differences between different training modalities. However, we encourage future researchers to refine the rubric to understand the generalizability and reliability of its results.

## Conclusions

In this randomized trial, VR training was found to be non-inferior to an e-module for asynchronous PPE training in a heterogeneous group of medical students, residents, and faculty. In residents alone, VR training led to improved PPE performance compared to the e-module training. Overall, VR participants felt more confident and prepared to don and doff PPE after training compared to their e-module counterparts. These findings are particularly relevant given the ongoing COVID-19 pandemic and the increasing need to provide effective PPE training to healthcare providers. Future studies should evaluate how to integrate the VR program into the medical curriculum and identify further subgroups that may benefit from the program. Additionally, long-term retention of VR donning and doffing PPE training must be monitored in the future to further pursue a golden standard for PPE training. 
